# Phage single-stranded DNA-binding protein or host DNA damage triggers the activation of the AbpAB phage defense system

**DOI:** 10.1128/msphere.00372-23

**Published:** 2023-10-26

**Authors:** Takaomi Sasaki, Saya Takita, Takashi Fujishiro, Yunosuke Shintani, Satoki Nojiri, Ryota Yasui, Tetsuro Yonesaki, Yuichi Otsuka

**Affiliations:** 1 Department of Biochemistry and Molecular Biology, Graduate School of Science and Engineering, Saitama University, Saitama, Japan; 2 Department of Biological Sciences, Graduate School of Science, Osaka University, Osaka, Japan; University of Michigan, Ann Arbor, Michigan, USA

**Keywords:** bacteriophages, *Escherichia coli*, phage defense system, AbpA-AbpB

## Abstract

Bacteria have developed various defense mechanisms against phages. Abortive infection (Abi), a bacterial defense mechanism, can be achieved through various means, including toxin-antitoxin systems, cyclic oligonucleotide-based antiphage signaling systems, and retrons. AbpA and AbpB (AbpAB) defend against many lytic phages harboring double-stranded DNA genomes in *Escherichia coli*; however, how AbpAB senses phage infection and inhibits its propagation remains unclear. Here, we demonstrated that AbpAB inhibited the growth of the φX174 lytic phage with single-stranded DNA (ssDNA) as well as the lysogenization and induction of the Sakai prophage 5 lysogenic phage. The AbpAB defense system limits T4 and φX174 phage propagation via Abi. AbpA contains a nuclease domain at its N-terminus, and AbpB has an ATP-dependent RNA helicase domain; both domains are required for phage defense. This system is activated by phage Gp32 binding to ssDNA and inhibits *E. coli* growth. Without phage infection, DNA replication inhibitors or defects in the DNA repair factors RecB and RecC activate this system. Therefore, the *E. coli* AbpAB defense system may sense DNA-protein complexes, including the phage-encoded ssDNA-binding protein or those formed by interrupting host DNA replication or repair.

**IMPORTANCE:**

Although numerous phage defense systems have recently been discovered in bacteria, how these systems defend against phage propagation or sense phage infections remains unclear. The *Escherichia coli* AbpAB defense system targets several lytic and lysogenic phages harboring DNA genomes. A phage-encoded single-stranded DNA-binding protein, Gp32, activates this system similar to other phage defense systems such as Retron-Eco8, Hachiman, ShosTA, Nhi, and Hna. DNA replication inhibitors or defects in DNA repair factors activate the AbpAB system, even without phage infection. This is one of the few examples of activating phage defense systems without phage infection or proteins. The AbpAB defense system may be activated by sensing specific DNA-protein complexes.

## INTRODUCTION

Bacteria have developed various defense mechanisms against phages for their survival and propagation. Bacteria prevent phage adsorption by altering or concealing phage receptors, inhibit phage propagation by cleaving phage nucleic acids through restriction-modification and the CRISPR-Cas system, or commit suicide before phage progeny is produced (1, 2). The defense mechanism involving suicide is termed abortive infection (Abi). As Abi leads to the premature death of a phage-infected cell before the phage completes its lifecycle, few phage progenies propagate, protecting the surrounding clonal bacteria from phage infections. Abi can be achieved through toxin-antitoxin (TA) systems, cyclic oligonucleotide-based antiphage signaling systems (CBASS), and retrons (1, 3–6). In TA-mediated Abi, phage infection triggers antitoxin loss or liberates toxins from the toxin-antitoxin complexes, resulting in toxin-mediated arrests of bacterial growth by inhibiting essential cellular processes, consequently inhibiting phage propagation (4, 7). In CBASS-mediated Abi, phage infection activates a cGAS/DncV-like nucleotidyltransferase (CD-NTase), generating cyclic oligonucleotides. These second messengers bind and activate various effector proteins, including phospholipase, endonuclease, and peptidase, causing bacterial cell death (5, 8). Retron-Eco48 and -Se72 are activated by inhibiting bacterial RecBCD after phage infection, causing cell growth arrest via unknown mechanisms (6, 9).

Recently, phage components that induce Abi by activating defense systems have been identified. Upon binding of the phage capsid protein Gp57 to CapRel, a member of the toxSAS TA family, the N-terminal toxin of CapRel is released from the C-terminal antitoxin, becoming activated (7, 10). Similarly, diverse phages evade *Pseudomonas aeruginosa* CBASS via mutations in their major capsid protein, suggesting that the capsid protein triggers CBASS activation (11). The single-stranded DNA (ssDNA)-binding protein (SSB) of the phage, which is critical for DNA replication, activates several defense systems, including the helicase/nuclease Hna proteins from *Sinorhizobium meliloti* and *Escherichia coli*, Retron-Eco8 from *E. coli*, and nuclease-helicase immunity (Nhi) from *Staphylococcus epidermidis* (9, 12, 13). Furthermore, the components of the phage core replication machinery, such as primase, DNA helicase, and DNA polymerase, activate many antiphage systems, including AbiQ, DarTG, Borvo, Lamassu, ietAS, and AbpAB (9). Although novel phage components that induce Abi have been identified, the molecular mechanisms underlying the activation of phage defense systems by these components remain unclear.

**Fig 1:**
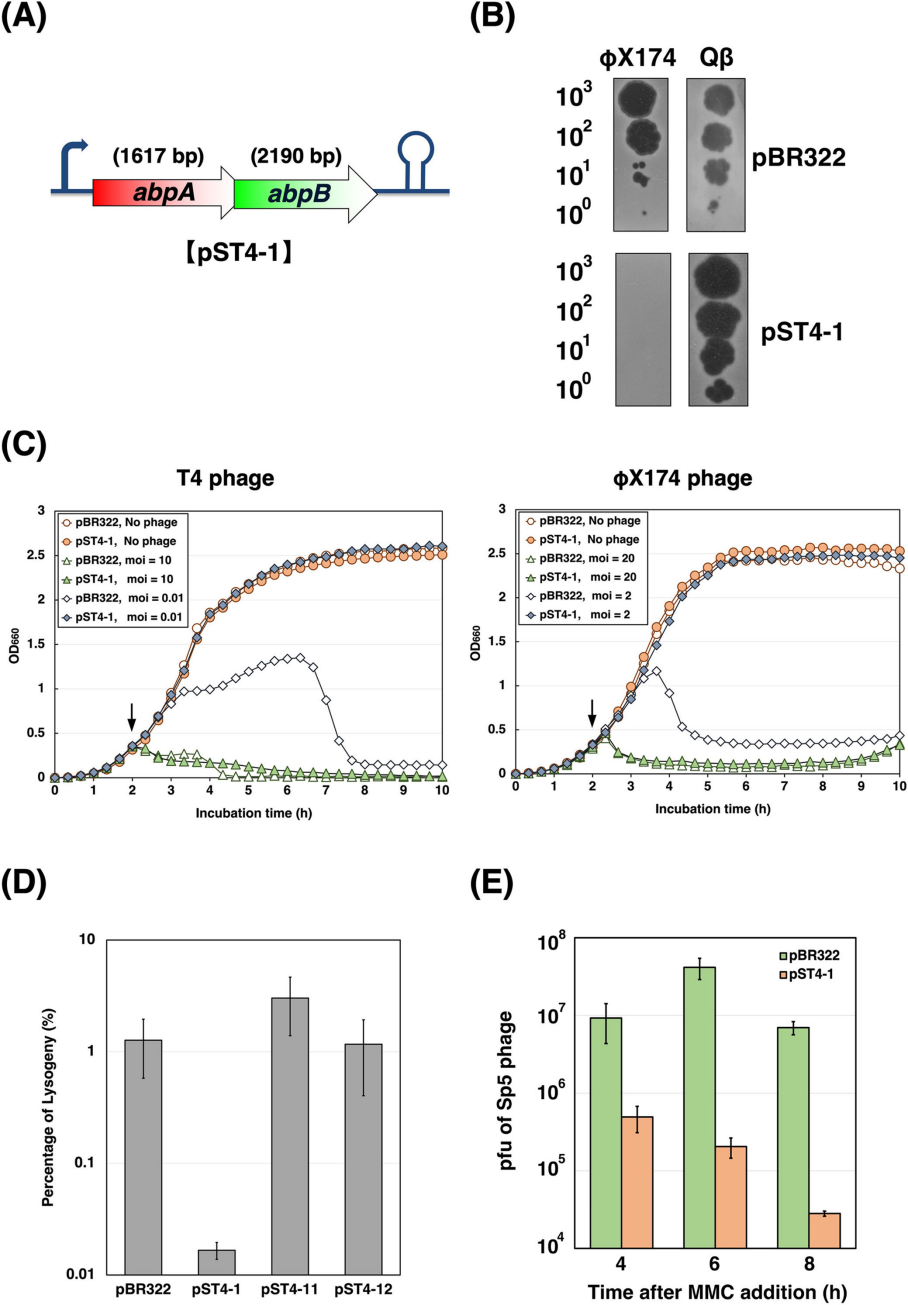
*Escherichia coli* AbpAB defends against many phages with DNA genomes. (**A**) Genetic organization of the *abpA-abpB* locus. The coding regions of *abpA* and *abpB* overlap by four nucleotides. (**B**) Effect of AbpAB expression on phage growth. Bacterial suspensions containing the number of φX174 or Qβ phage particles indicated on the left were spotted onto plates seeded with the *E. coli* C or A/λ strain harboring pBR322 or pST4-1. The plates were incubated overnight at 30°C. (**C**) Growth of *E. coli* cells expressing AbpAB after T4 or φX174 infection. *E. coli* K-12 strain TY0807 or C strain cells harboring pBR322 or pST4-1 were infected with phage T4 (left) or φX174 (right) at an MOI of 0, 0.01, 2, 10, or 20 when the optical density at 660 nm (OD_660_) reached approximately 0.3 (arrow). (**D**) Effect of AbpAB expression on Sakai prophage 5 (Sp5) lysogeny. TY0807 cells harboring pBR322, pST4-1, pST4-11, or pST4-12 were infected with Sp5 lysogenic phages containing the KM-resistance gene and Sp5-lysogenized cells were selected on LB-agar plates supplemented with KM. The lysogeny percentage was calculated by dividing the number of lysogenized cells by the total recipient cells. (**E**) Effect of AbpAB expression on Sp5 induction. MG1655-Sp5 (km^r^) harboring pBR322 or pST4-1 were grown and treated with MMC for 4, 6, or 8 h. Sp5 phages released were plated with MG1655 cells to measure the number of plaque-forming units (pfu). The pfu data represent the mean ± standard deviation of values from at least triplicate measurements.

The *abpA* and *abpB* genes in the *E. coli* K-12 genome form an operon, and the coding regions of both genes overlap by four nucleotides (Fig. 1A). AbpA and AbpB (AbpAB) coexpression suppresses the growth of many lytic phages harboring double-stranded DNA (dsDNA) genomes (14). AbpA and AbpB physically interact for phage defense. AbpAB is activated after the middle stage of infection and inhibits DNA replication in T2, T4, and T7 phages. However, the T4 phage can evade the AbpAB defense system through a mutation in gene 41, which encodes a replicative DNA helicase (14, 15). However, the function of Gp41 in this system, the AbpAB activation mechanism after phage infection, and how AbpAB inhibits phage propagation remain unclear.

In this study, we aimed to examine the effect of AbpAB on the growth of lytic phages harboring ssDNA or RNA as a genome and a lysogenic phage and to identify the AbpA and AbpB domains required for phage defense. This study also aimed to determine whether phage DNA replicative components trigger the AbpAB defense system activation. Finally, we aimed to identify new conditions under which the AbpAB system is activated without phage infection.

## RESULTS

### AbpAB defends against many phages with DNA genomes

We assessed the impact of AbpAB on the growth of lytic phages containing ssDNA or RNA, specifically φX174 or Qβ phages. Both phages grew in cells harboring the empty plasmid pBR322 (Fig. 1B). Qβ grew normally in cells harboring the pST4-1 plasmid expressing AbpA and AbpB; however, the efficiency of plating (EOP) of φX174 decreased to <10^−4^, indicating that AbpAB inhibits φX174 growth. We also examined whether the AbpAB defense system induces Abi. Cells harboring pBR322 or pST4-1 were infected with T4 or φX174 phages at a low or high multiplicity of infection (MOI). Cells harboring pBR322 grew slowly until approximately 6 h post-T4 infection and underwent cell lysis at a low MOI, whereas cells harboring pST4-1 exhibited normal growth (Fig. 1C). Similar growth curves after infection at a low MOI were observed for φX174. T4 or φX174 infection at a high MOI caused immediate growth arrest and lysis in cells harboring pBR322 or pST4-1; however, the collapse of cells harboring pST4-1 after T4 infection occurred slightly earlier than the phage-induced lysis in cells harboring pBR322. Therefore, AbpAB defends against T4 and likely φX174 via Abi rather than a direct phage interference, such as restriction-modification or CRISPR-Cas (9).

We then investigated the effects of AbpAB on lysogenization and induction of lysogenic phages. The lambdoid Sp5 (Sakai prophage 5), a prophage of the enterohemorrhagic *E. coli* O157:H7 Sakai strain, can infect non-pathogenic *E. coli* K-12 strains (16). We first assessed the lysogenization rates of the Sp5 phage in cells expressing AbpAB, AbpA, or AbpB (Fig. 1D). *E. coli* K-12 TY0807 cells harboring each plasmid were infected with an equal number of Sp5 phages containing a kanamycin (KM)-resistance gene. Lysogenizing the Sp5 phage transformed TY0807 cells into KM-resistant cells. Lysogenization in cells harboring pBR322 was 1.3%, whereas that in cells with pST4-1 was considerably reduced to 0.017%. No reduction in the lysogenization rate in cells with pST4-11 expressing AbpB or pST4-12 expressing AbpA was observed. These results indicate that AbpA and AbpB coexpression inhibited Sp5 phage lysogenization.

We then examined the effect of AbpAB on Sp5 prophage induction. The Sp5 prophage was induced in MG1655-Sp5 (km^r^) cells harboring pBR322 or pST4-1 using mitomycin C (MMC), a DNA-damaging agent that crosslinks two DNA bases and triggers prophage induction (17). The number of Sp5 phage progeny released from cells was then measured using a plaque-forming assay (Fig. 1E). Compared to the phage progeny from cells harboring pBR322, the number of phage progeny from cells harboring pST4-1 at 4, 6, and 8 h after MMC addition was reduced to <1% of the control, indicating that AbpAB inhibits Sp5 prophage induction. Therefore, AbpAB defends against many lytic phages with DNA genomes and lysogenic phages.

### Nuclease domain at the AbpA N-terminus is required for phage defense

**Fig 2:**
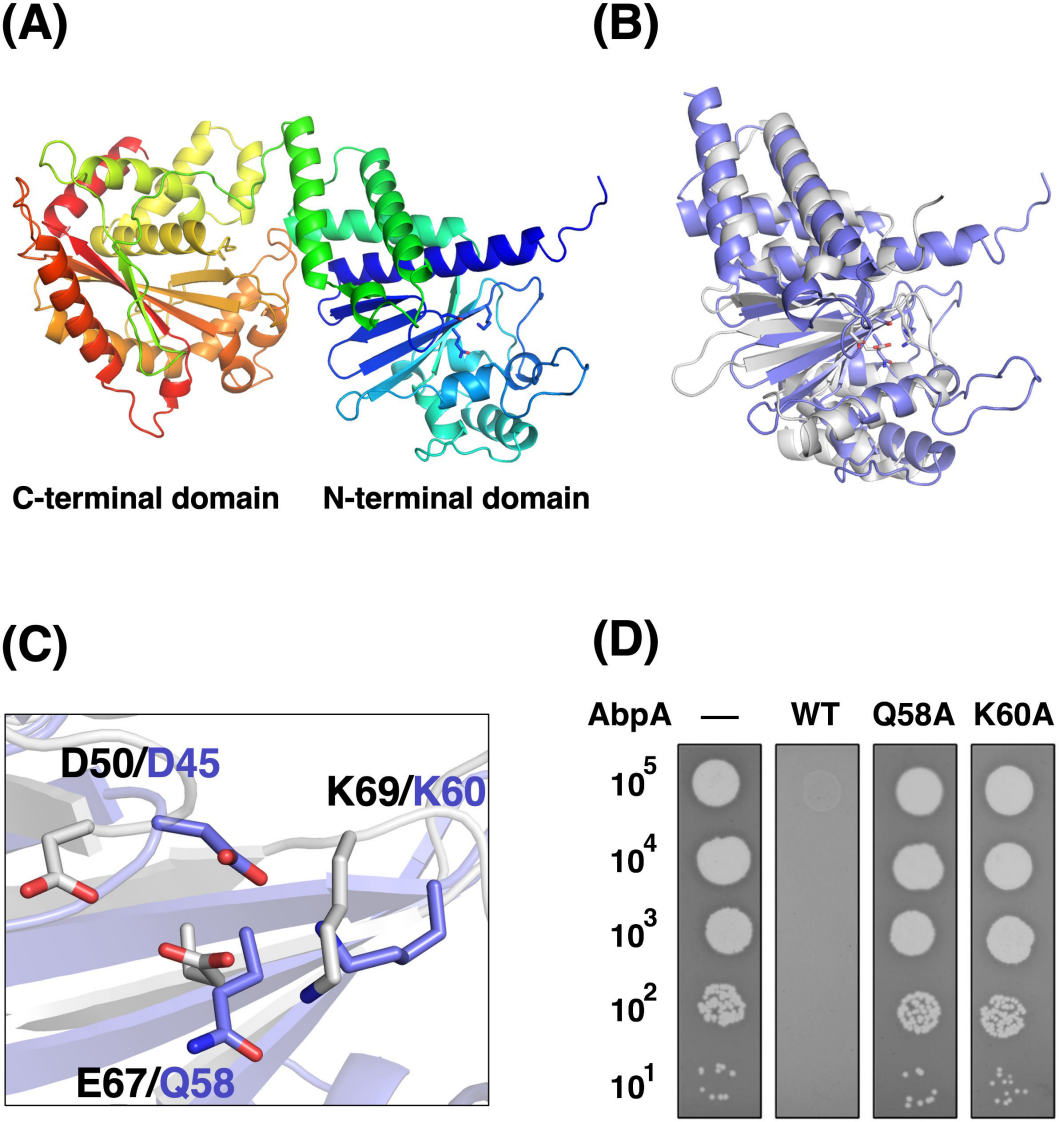
Nuclease domain at the N-terminus of AbpA is required for phage defense. (**A**) An AlphaFold2 model of AbpA. The polypeptide of AbpA is rainbow-colored from the N-terminus (blue) to the C-terminus (red). The Asp45, Gln58, and Lys69 side chains are shown as stick models. (**B**) Superimposition of the N-terminal domain of the AbpA model (slate blue) on the crystal structure of CD-NTase-associated protein 4 (Cap4) (white) (PDB ID: 6WAN). The C-terminal domain of AbpA is omitted for clarity. (**C**) Close-up view of a putative active site of AbpA superimposed on Cap4. Residues at the equivalent positions are labeled (slate blue: AbpA, black: Cap4). (**D**) T4 phage growth on cells expressing AbpA mutants and AbpB. A suspension containing the number of T4 phage particles indicated on the left was applied as spots onto plates seeded with TY0807 cells harboring pBR322 (–), pST4-1 (WT), pST4-13 (Q58A), or pST4-14 (K60A). The plates were incubated overnight at 30°C.

AbpA encodes a 538-amino acid (aa)-long protein and contains two predicted Pfam domains—the CD-NTase-associated protein 4 (Cap4) dsDNA endonuclease (PF14130; aa 5-215) and HamA (PF08878; aa 294–519). Cap4 contains an N-terminal effector domain with dsDNA nuclease activity and a C-terminal SAVED domain to sense secondary nucleotide messengers synthesized after phage infection, functioning in the cyclic oligonucleotide-based antiphage signaling systems (CBASS) (18). The Cap4 endonuclease domain shares structural homology with type II restriction endonucleases. HamA is an uncharacterized protein that participates in the Hachiman antiphage system with HamB (19, 20). We obtained the AlphaFold2 model of AbpA from the AlphaFold Protein Structure Database (Fig. 2A) and searched for proteins similar to the AbpA model. The N-terminal region of AbpA showed high structural similarity to the dsDNA endonuclease domain of Cap4 (Fig. 2B). Cap4 endonuclease activity requires Asp50, Glu67, and Lys69, which coordinate with divalent metal ions (18). In AbpA, the Asp45, Gln58, and Lys60 residues occupy the equivalent steric position as the three amino acids required for Cap4 endonuclease activity (Fig. 2C). We constructed two plasmids expressing AbpA mutants wherein Gln58 or Lys60 was substituted with alanine to clarify whether these amino acids are required for the AbpAB defense system. AbpB and AbpA (Q58A) or AbpA (K60A) coexpression allowed normal T4 phage growth (Fig. 2D), indicating the significance of both amino acids in phage defense. This finding suggests that the DNA endonuclease activity of AbpA is essential in the defense system.

### ATP-dependent RNA helicase domain of AbpB is required for phage defense

**Fig 3:**
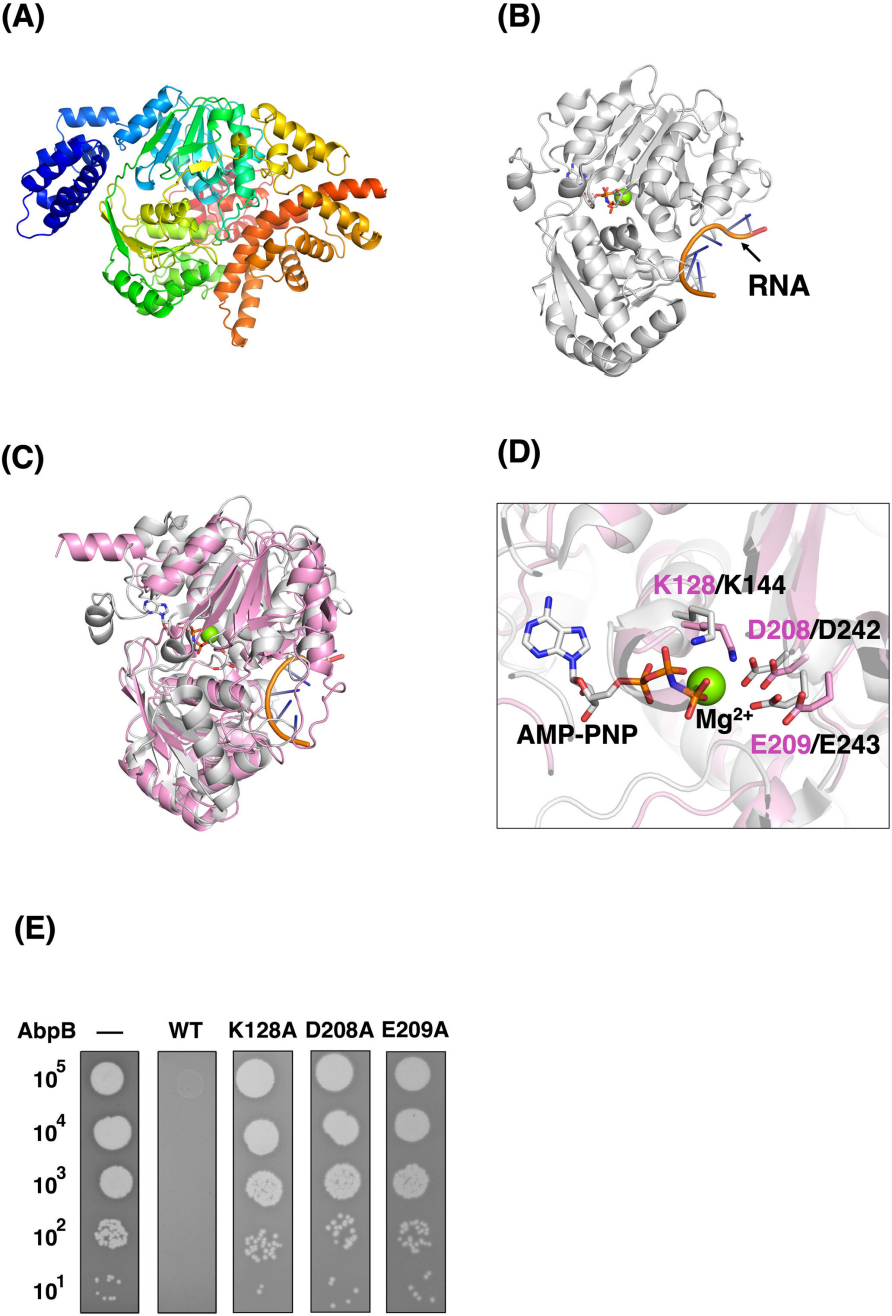
ATP-dependent RNA helicase domain of AbpB is required for phage defense. (**A**) An AlphaFold2 model of AbpB. The AbpB polypeptide is rainbow-colored from the N-terminus (blue) to the C-terminus (red). (**B**) The crystal structure of human RNA helicase Dbp5 (DDX19B) (PDB ID: 3FHT) is partially homologous to AbpB. A black arrow indicates RNA bound to DDX19B. An ATP analog (AMP-PNP) and Mg^2+^ are shown in stick and sphere models, respectively. (**C**) Superimposition of the middle domain of the AbpB (pink) model on DDX19B (white). The N-and C-terminal domains of AbpB are omitted for clarity. (**D**) Close-up view of a putative ATP-binding region of AbpB superimposed on DDX19B. Residues at the equivalent positions are shown in stick models with labels (pink: AbpB, black: DDX19B). (**E**) Growth of T4 phages on cells expressing AbpA and AbpB mutants. A suspension containing the number of T4 phage particles indicated on the left was applied as spots onto plates seeded with TY0807 cells harboring pBR322 (–), pST4-1 (WT), pST4-15 (K128A), pST4-16 (D208A), or pST4-17 (E209A). The plates were incubated overnight at 30°C. The data concerning TY0807 cells harboring pBR322 (–) or pST4-1 (WT) are being reused from Fig. 2D because they were the same internally controlled experiment.

AbpB encodes a 729-aa-long protein and contains a helicase-conserved C-terminal domain (PF00271; aa 364–433). We obtained the AbpB AlphaFold2 model (Fig. 3A) and identified several ATP-dependent RNA helicases showing high structural similarity with *Z*-scores > 20. Human RNA helicase Ddp5 (DDX19B) structure (Fig. 3B) (21) exhibited high similarity to the middle domain structure of AbpB (Fig. 3C). DDX19B belongs to the DEAD (Asp-Glu-Ala-Asp)-box RNA helicase family, which unwinds RNA duplexes or displaces bound protein complexes in an ATP-dependent manner (22). Lys128, Asp208, and Glu209 in AbpB occupy the equivalent steric position to the amino acids (Lys144, Asp242, and Glu243) required for ATP binding to DDX19B (Fig. 3D). We constructed plasmids expressing AbpB mutants wherein these amino acid residues were substituted with alanine. Coexpression of AbpA and each AbpB mutant did not inhibit T4 phage growth (Fig. 3E), confirming that these amino acids are essential for phage defense. These results suggest that the AbpB helicase activity is vital for bacterial defense.

### AbpAB defense system is activated by phage-encoded proteins bound to ssDNA

**Fig 4:**
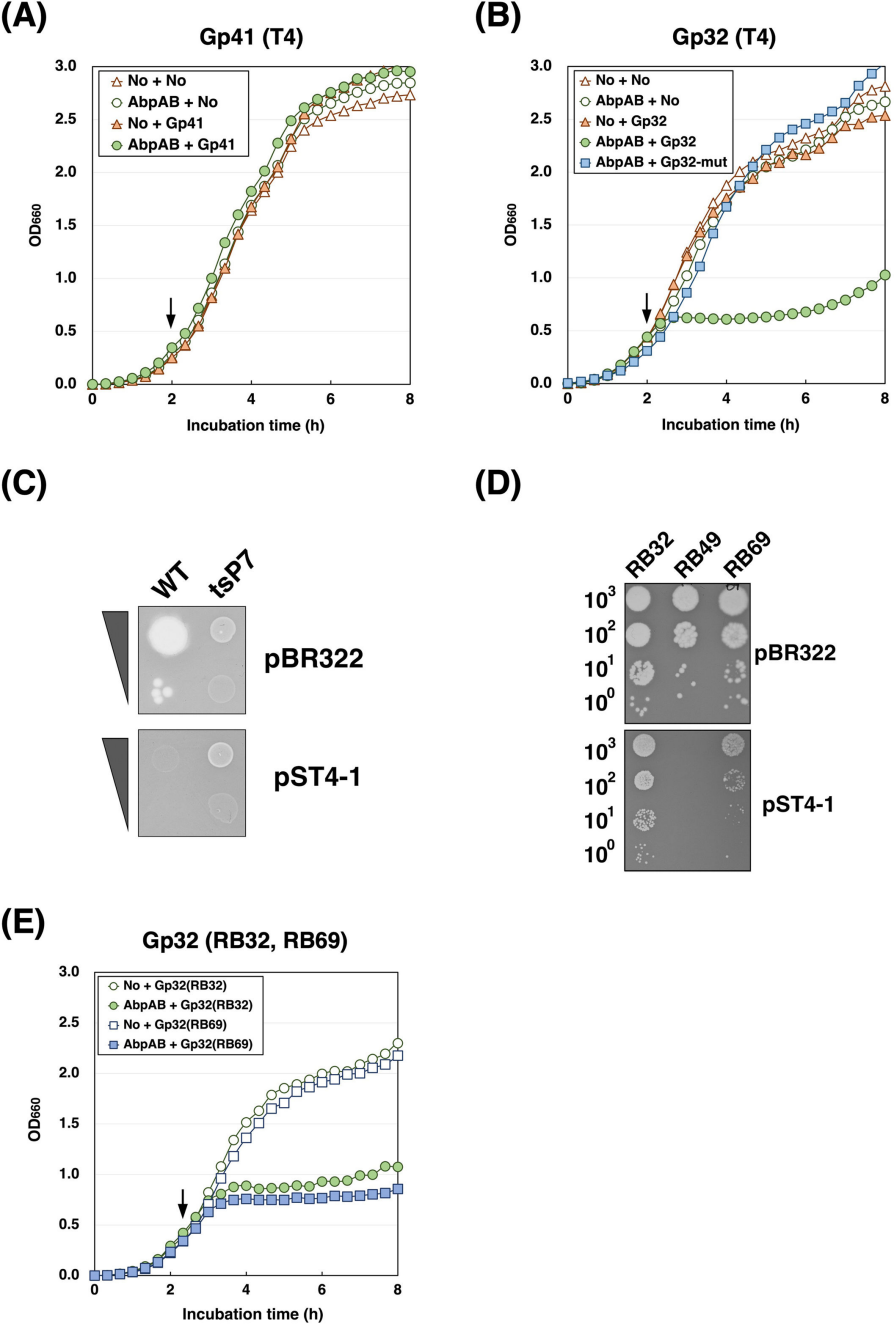
AbpAB defense system is activated by phage-encoded proteins bound to ssDNA. (**A**) Effect of T4 Gp41 on the growth of *E. coli* cells expressing AbpAB. TY0807 cells harboring pBR322 and pBAD33 (No + No), pST4-1 and pBAD33 (AbpAB + No), pBR322 and pBAD33-gp41 (No + Gp41), or pST4-1 and pBAD33-Gp41 (AbpAB + Gp41) were treated with L-ara when the OD_660_ reached approximately 0.3. (**B**) Effect of T4 Gp32 on the growth of *E. coli* cells expressing AbpAB. TY0807 cells harboring pBR322 and pBAD33 (No + No), pST4-1 and pBAD33 (AbpAB + No), pBR322 and pBAD33-gp32 (No + Gp32), pST4-1 and pBAD33-Gp32 (AbpAB + Gp32), or pST4-1 and pBAD33-gp32 (C87A C90A) (AbpAB + Gp32-mut) were treated with L-ara when the OD_660_ reached approximately 0.4. (**C**) Effect of AbpAB expression on the growth of T4 mutants of gene *32*. A suspension containing T4 wildtype or *tsP7* (*ts* mutant of gene *32*) at a 1:10 dilution was spotted onto plates seeded with TY0807 cells harboring pBR322 or pST4-1. The plates were incubated overnight at 30°C. (**D**) Effect of AbpAB expression on the growth of RB phages. A suspension containing the number of RB23, RB49, or RB69 phage particles indicated on the left was spotted onto plates seeded with TY0807 cells harboring pBR322 or pST4-1. The plates were incubated overnight at 30°C. (**E**) Effect of the Gp32 of RB32 and RB69 phages on the growth of *E. coli* cells expressing AbpAB. TY0807 cells harboring pBR322 and pBAD33-gp32(RB32) [No +Gp32(RB32)], pST4-1 and pBAD33-gp32(RB32) [AbpAB + Gp32(RB32)], pBR322 and pBAD33-gp32(RB69) [No + Gp32(RB69)], or pST4-1 and pBAD33-gp32(RB69) [AbpAB + Gp32(RB69)] were treated with L-ara when the OD_660_ reached approximately 0.4.

The T4 phage can evade the AbpAB defense system through a mutation in gene 41, which encodes a replicative DNA helicase, indicating the need for Gp41 (14). We constructed the plasmid pBAD33-Gp41, which contained gene 41 under an arabinose-inducible promoter, to investigate whether Gp41 could activate the AbpAB system and induce cell growth arrest. Unexpectedly, cells harboring pST4-1 and pBAD33-gp41 grew normally after L-arabinose (L-ara) addition (Fig. 4A), indicating that Gp41 is not sufficient to activate this system.

Coexpressing several phage defense systems with phage SSB inhibits bacterial cell growth (9, 12). Therefore, we investigated whether the T4 phage SSB, Gp32, could induce cell growth arrest in the presence of the AbpAB defense system. Whereas Gp32 expression in cells harboring pBR322 had no impact on cell growth, cells harboring pST4-1 exhibited immediate growth retardation upon Gp32 expression (Fig. 4B). We examined the growth of a T4-mutant phage (tsP7) with a missense mutation in gene *32* to validate this further (23). Wild-type phages did not grow on cells carrying pST4-1; however, tsP7 phages showed growth on these cells comparable to that on cells carrying pBR322 (Fig. 4C). These results indicated that Gp32 activated this system to inhibit cell growth. The effect of Gp32 on the AbpAB defense system depended on AbpA and AbpB coexpression (Fig. S1A). Subsequently, we examined whether activating this system through Gp32 requires the ability of Gp32 to bind ssDNA. Gp32 binds to ssDNA through its Zn-finger domain, a DNA-binding motif (24). The Cys77, Cys87, Cys90, and His64 residues in the Zn-finger are required for ssDNA binding. We constructed a plasmid expressing a Gp32 mutant wherein Cys87 and Cys90 residues were substituted with alanines. Coexpressing the Gp32 mutant with AbpAB did not retard cell growth (Fig. 4B), suggesting that activating this system requires Gp32 binding to ssDNA.

We previously investigated phages capable of evading this system and identified T4-like phages RB32 and RB69. RB32 and RB69 could grow in cells expressing AbpAB, whereas RB49 could not (Fig. 4D). However, the plaque sizes of RB32 and RB69 became smaller by expressing AbpAB, indicating that the AbpAB system was slightly active. To support this, coexpressing Gp32 from RB32 or RB69 with AbpAB induced cell growth retardation (Fig. 4E). These results strongly suggest that both phages possess factors that inhibit the AbpAB system once it is activated post-infection.

### AbpAB is activated by interrupting host DNA replication or repair without phage infection

**Fig 5:**
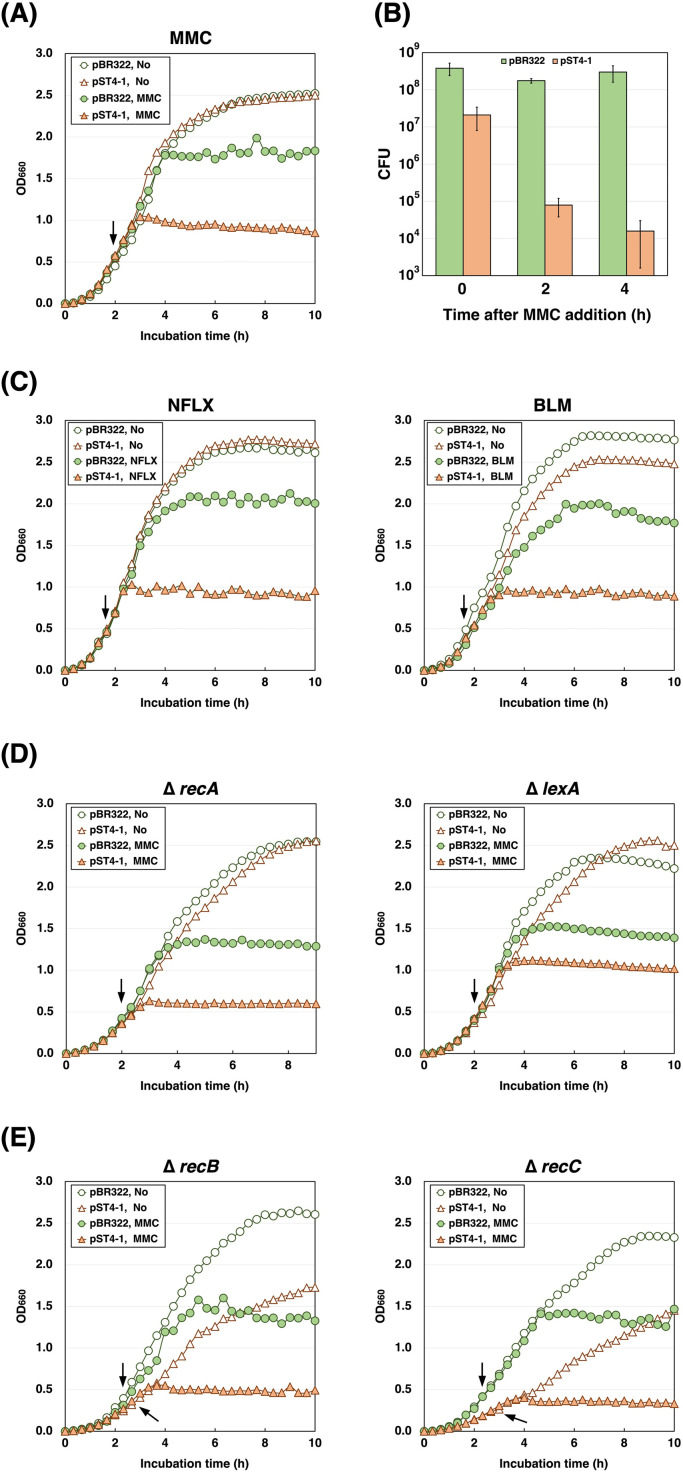
AbpAB is activated by inhibiting host DNA replication or repair. (**A**) Effect of MMC on the growth of *E. coli* cells expressing AbpAB. TY0807 cells harboring pBR322 or pST4-1 were treated with or without MMC when the OD_660_ reached approximately 0.5. (**B**) Effect of MMC on the viability of *E. coli* cells expressing AbpAB. TY0807 cells harboring pBR322 or pST4-1 were grown until the OD_660_ reached 0.3–0.5 and then treated with MMC for 0, 2, and 4 h. The colony-forming unit data represents the mean ± standard deviation of at least triplicate measurements. (**C**) Effect of DNA synthesis inhibitors on the growth of *E. coli* cells expressing AbpAB. TY0807 cells harboring pBR322 or pST4-1 were treated with or without NFLX (left) or BLM (right) when the OD_660_ reached approximately 0.5. (**D**) Effect of MMC on the growth of *E. coli* ∆*recA* or ∆*lexA* cells expressing AbpAB. JW2669 (∆*recA*) (left) or ME9628 (∆*lexA*) (right) cells harboring pBR322 or pST4-1 were treated with or without MMC when the OD_660_ reached approximately 0.4. (**E**) Effect of AbpAB on the growth of *E. coli* ∆*recB* or ∆*recC* cells. JW2788 (∆*recB*) (left) or JW2790 (∆*recC*) (right) cells harboring pBR322 or pST4-1 were treated with or without MMC when the OD_660_ reached approximately 0.4.

We sought to identify conditions under which the AbpAB defense system becomes active without phage infection. AbpAB does not affect *E. coli* growth under normal growth conditions (14) (Fig. 5A). When MMC was added to cells harboring pBR322 or pST4-1 at sub-inhibitory concentrations, the cells harboring pBR322 continued to grow although minor growth inhibition was observed (Fig. 5A). Conversely, cells with pST4-1 exhibited substantial growth retardation. The number of colony-forming units (CFU) indicated that the viable cell count with pST4-1 was reduced to <1% after 2 and 4 h of MMC addition when compared to that before MMC addition (Fig. 5B). These results suggest that MMC activates the AbpAB defense system and induces cell growth arrest and death. Subsequently, we explored whether other DNA replication inhibitors could activate this system. When we added norfloxacin (NFLX), a DNA gyrase inhibitor, or bleomycin (BLM), which cleaves DNA, cells with pST4-1 exhibited noticeable growth retardation, similar to the effect of MMC treatment (Fig. 5C). However, when we added hydroxyurea, 5-azacytidine, and streptomycin, which inhibit deoxyribonucleotide biosynthesis, cytosine DNA methylation, and translation, respectively, no growth retardation occurred (Fig. S2). These results suggest that the interruption of replication that occurs on host DNA activates the AbpAB defense system.

DNA replication inhibitors such as MMC and BLM induce SOS responses. ssDNA and *E. coli* SSB production increases during the SOS response (25). Therefore, we examined whether *E. coli* SSB inhibited the growth of AbpAB-expressing cells, as did T4 Gp32. *E. coli* SSB and AbpAB coexpression did not exhibit growth retardation (Fig. S1B). In addition, we examined whether RecA and LexA, two vital factors in the SOS response, contribute to MMC-induced AbpAB defense system activation. When MMC was added to ∆*recA* or ∆*lexA* cells harboring pBR322, the optical density at 660 nm (OD_660_) value corresponding to the steady state was lower than that of cells without MMC (Fig. 5D), possibly because the SOS response or DNA repair mechanisms did not work. The growth of *recA* or *lexA*-deleted cells with pST4-1 was retarded after MMC addition, suggesting that RecA and LexA are not involved in the MMC-induced activation of this system. RecB and RecC are proteins in the heterotrimeric helicase/nuclease complex known as RecBCD, which promotes homologous recombination repair in dsDNA breaks (26). ∆*recB* or ∆*recC* cells harboring pBR322 grew substantially slower than wild-type cells (Fig. S3), possibly owing to their defective DNA repair capacity (27). Both deletion cells harboring pST4-1 exhibited evident growth retardation after MMC addition (Fig. 5E), indicating that RecB and RecC are not essential in MMC-induced AbpAB defense system activation. However, ∆*recB* or ∆*recC* cells harboring pST4-1 grew considerably slower than did those harboring pBR322 without MMC (Fig. 5E; Fig. S3), indicating that AbpAB is partially activated by *recB* or *recC* deletion, resulting in inhibiting cell growth. These findings suggest that the AbpAB defense system can be triggered by interrupting host DNA replication or repair without phage infection.

## DISCUSSION

This study investigated the activation of the AbpAB defense system and its role in inhibiting phage propagation. The SSB Gp32 of the T4, RB32, and RB69 phages activates AbpAB to inhibit cell growth, thereby inducing Abi. Retron-Eco8 is activated by the T7 SSB or SECphi4 phages and induces cell growth inhibition (9). Similarly, coexpressing the Hachiman defense system with phage SSBs inhibits cell growth (9). Hna, a protein containing helicase and nuclease domains, is also activated by phage SSB in Abi (12), and phage SSB activates the Nhi defense system (13). Therefore, phage SSBs are vital activators of many phage defense systems. Binding T4 Gp32 to ssDNA is required for the AbpAB defense system, indicating the importance of ssDNA as well as Gp32 in activating this system. In AbpAB-expressing cells, T4 early and middle proteins were synthesized normally, whereas late proteins were hardly synthesized (14). This result indicates that the AbpAB defense system is activated after the middle stage of T4 infection, as evidenced by the increase in ssDNA and Gp32 levels at the middle stage when DNA replication occurs. However, how Gp32 and ssDNA activate this system remains unclear. Gp32 primarily binds to the ssDNA produced during phage DNA replication; therefore, the DNA replicative protein complex containing Gp32 and possibly Gp41 could activate the AbpAB defense system. A proposed model suggests that Nhi is activated by the DNA replicative protein complex, including phage SSB, causing phage DNA degradation (13). Similarly, Hachiman and ShosTA may respond to a protein-DNA complex formed as an intermediate during phage DNA replication or recombination (9).

Although a missense mutation in gene 41 allowed T4 phages to evade the AbpAB defense system (14), Gp41 expression alone did not inhibit AbpAB-expressing cell growth, suggesting that Gp41 may activate this system through Gp32 in T4-infected cells. As a mutation in gene 41 encoding a DNA helicase inhibits DNA unwinding, the amount of ssDNA should be decreased. This decrease could reduce the ssDNA-Gp32 complex levels, possibly preventing activating this system. T4 Gp32 shares a high homology of 98% with Gp32 from RB32 and 90% with Gp32 from RB69. The conserved Cys87 and Cys90 residues in a Zn-finger motif among these three phages indicate that Gp32 from RB32 or RB69 is possibly capable of binding to ssDNA and activating the AbpAB defense system. Notably, even though these Gp32 inhibited the growth of cells expressing AbpAB, RB32 and RB69 phages escaped this system and grew. Therefore, both phages must be equipped with factors that inhibit the AbpAB system. Identifying and characterizing these inhibitory factors can provide insight into regulating the AbpAB defense system between bacteria and phages.

We demonstrated that interrupting host DNA replication or repair activated the AbpAB system without phage infection. This is one of the few examples of phage defense system activation without phage infection or phage-related proteins. A similar concept was recently shown in CBASS, seemingly activated following anti-folate antibiotic treatment (28, 29). As MMC, BLM, and NFLX act directly on the host DNA, AbpAB possibly senses the DNA-protein complexes formed through interrupted host DNA replication. The SOS response factors (RecA and LexA), *E. coli* SSB, and DNA repair factors (RecB and RecC) were not involved in the MMC-induced AbpAB defense system activation. However, AbpAB seems to be partially activated by *recB* or *recC* deletion. The DNA-protein intermediate complexes formed during DNA repair accumulate in these repair-deficient mutants. Hence, AbpAB may be activated by sensing the presence or increased levels of these intermediate DNA-protein complexes.

The AbpAB system prevented the propagation of the φX174 phage with ssDNA genomes. This phage encodes 11 genes, 4 of which (A, A*, C, and J) encode proteins involved in DNA replication or binding. Proteins C and J expressed from the plasmid did not trigger AbpAB-mediated cell growth inhibition (S. Takita and Y. Otsuka, unpublished data). We have also investigated the ability of the A and A* proteins to activate the AbpAB system. As the Qβ RNA phage encodes only four proteins, which are unlikely to bind to ssDNA or directly inhibit host DNA replication, AbpAB showed no defense activity against the Qβ phage. Additionally, AbpAB inhibited the lysogenization and induction of the Sp5 phage; however, the underlying molecular mechanisms remain unclear. In the induction experiment, MMC was used as a DNA-damaging agent. MMC might have activated the AbpAB defense system and inhibited cell growth and viability, causing a decrease in the induced phage progenies. Another possibility is that an uncharacterized Sp5 SSB may activate this system. In the context of AbpAB-mediated inhibition of Sp5 lysogenization, cell suicide following infection may result in a lower lysogeny rate. We examined the lytic propagation of Sp5 phages to verify this hypothesis. However, no significant EOP reduction by AbpAB expression was observed (Fig. S4), likely dismissing the abovementioned hypothesis. During lysogenization, the host genome is cleaved, and an integrase inserts the phage genome. The AbpAB defense system may then be activated, inhibiting cell growth and viability and decreasing the lysogenization rate.

We identified specific domains in AbpA and AbpB required for phage defense; however, the biochemical activities of both proteins remain unestablished. The N-terminal region of AbpA exhibits a high structural similarity to the dsDNA endonuclease domain of Cap4. As mutational analysis suggests the essentiality of DNA nuclease activity, AbpA could inhibit cell growth by cleaving host DNA. Similarly, many ATP-dependent RNA helicases exhibited high structural similarity to the AbpB model structure. Amino acid substitutions in the ATP-binding site abolished AbpAB defense activity, suggesting that the AbpAB defense system requires AbpB helicase activity. While helicases are involved in many defense systems, such as Hachiman, Hna, and Nhi, how they function in the defense systems and whether they target RNA or DNA molecules remain unclear. Several studies have suggested a potential relationship between the AbpAB and the Hachiman defense system: AbpA contains the HamA domain (previously known as DUF1837), and phage-encoded SSB can activate the AbpAB system, similar to recent findings for Hachiman (9). However, DELTA-BLAST (BLASTp) and Clustal Omega analyses demonstrated that the alignments between AbpA and HamA, or AbpB and HamB, were possible only in very limited regions, with the proteins sharing an identity of approximately 16%. Additionally, AbpA possesses the Cap4 nuclease domain required for antiphage function, whereas HamA does not. Therefore, we speculate that the AbpAB system is not a direct equivalent of the Hachiman system.

Numerous recently discovered phage defense systems have shown a considerably larger variety and number of defense systems than initially expected. Although most defense factors inhibit phage propagation when expressed in multi-copy plasmids, few studies have been conducted to determine whether defense factors expressed from the genome exhibit defense effects. When AbpAB was expressed from a plasmid, the EOP of the T4 phage was decreased to 10^−4^. However, in wild-type and *abpAB*-deleted cells, the EOP was comparable, even though AbpAB expressed from the genome reduced the burst size by approximately 10% (14). Therefore, examining the protective potential of various defense factors expressed from the genome against phages is crucial. As each defense system alone may not inhibit phage propagation, bacteria may have evolved various defense systems.

## MATERIALS AND METHODS

### *E. coli* strains and phages

All *E. coli* strains used in this study are listed in Table 1. Prof. Sekine of Rikkyo University kindly provided MG1655 and MG1655-Sp5 (Km^r^) cells. MG1655-Sp5 (Km^r^) does not produce the Shiga toxin 2 because of the *stx2A-stx2B* deletion. The *E. coli* A/λ and C strains were provided by Prof. Kashiwagi of Hirosaki University and Prof. Inagaki of Mie University, respectively. BW25113, JW2669, JW2788, JW2790, and ME9628 cells were provided by the National BioResource Project (National Institute of Genetics, Japan). The wild-type bacteriophages T4D, RB32, RB49, and RB69 were used (30). *tsP7* is a T4 phage with a temperature-sensitive mutation in gene *32* (23). Phages Qβ (31) and φX174 (NBRC 103405) were provided by Prof. Kashiwagi of Hirosaki University and Prof. Inagaki of Mie University, respectively.

**TABLE 1:** *Escherichia coli* strains used in this study

Strain	Genotype	Source/ or reference
TY0807	*sup^0^ araD139 hsdR* ∆*lacX74 rpsL araD^+^*	(4)
BW25113	*rrnB3* ∆*lacZ4787 hsdR514* ∆(*araBAD)567* ∆(*rhaBAD)568 rph-1*	NBRP-*E. coli* at NIG
JW2669	BW25113 ∆*recA*::*km^r^*	NBRP-*E. coli* at NIG
JW2788	BW25113 ∆*recB*::*km^r^*	NBRP-*E. coli* at NIG
JW2790	BW25113 ∆*recC*::*km^r^*	NBRP-*E. coli* at NIG
ME9628	*F^−^* ∆*lacX74 strA araD139* ∆(*ara leu)7697 galU^−^ galK^−^ hsr^−^ hsm^+^ lexA1* (*lnd^−^*) *zjb564::Tn10*	NBRP-*E. coli* at NIG
MG1655	λ^−^ *ilvG^−^ rfb-50 rph-1*	(32)
MG1655-Sp5 (km^r^)	MG1655-Sp5 (∆*stx2AB*::*km^r^*)	(32)
A/λ	*Su^+^ F^+^* λ resistant	(33)
C	NBRC 13898 (ATCC 13706)	NBRC

### Plasmid construction

The primers used in this study are listed in Table 2. A 4,512 bp DNA fragment of the T4 phage genome (2,760,763–2,765,275 of GenBank accession no. NC_000913.3) encoding *abpA*, *abpB*, and a part of *yfjJ* was ligated into the *Hin*dIII site of pBR322 (14). All restriction enzymes used in this study were purchased from Toyobo (Osaka, Japan) and Takara Bio (Shiga, Japan) for plasmid construction. pST4-1 was digested with *Sph*I or *Sac*I and self-ligated to generate pST4-11 or pST4-12. To generate pBAD24-gp32 or pBAD24-gp41, a DNA fragment containing gene *32* or *41* was amplified via PCR using the genomic DNA of the T4 phage as a template, and the primers YO-899 and YO-912 or YO-819 and YO-820, respectively. The PCR products were digested with *Eco*RI and *Hin*dIII or *Kpn*I and *Xba*I and ligated into the corresponding pBAD24 sites (34). pBAD24-gp32 or pBAD24-gp41 was then digested with *Bam*HI and *Hin*dIII or *Bam*HI and *Xba*I, respectively, and ligated into pBAD33 to construct pBAD33-gp32 or pBAD33-gp41, respectively. pBAD24-gp32(RB32) or pBAD24-gp32(RB69) was generated by amplifying a DNA fragment containing gene *32* via PCR using the genomic DNA of the RB32 or RB69 phages as a template and YO-929 and YO-930 or YO-931 and YO-932 as primers, respectively. The PCR products were digested with *Eco*RI and *Pst*I and ligated into the corresponding pBAD24 sites. pBAD24-gp32(RB32) or pBAD24-gp32(RB69) was then digested with *Bam*HI and *Pst*I and ligated into pBAD33 to construct pBAD33-gp32(RB32) or pBAD33-gp32(RB69), respectively. To generate pBAD33-ssb, a DNA fragment containing *ssb* was amplified through PCR using the genomic DNA of the *E. coli* K-12 strain as a template, and the primers YO924 and YO-925, digested with *Sal*I and *Sph*I, and ligated into pBAD33. Plasmids expressing AbpA, AbpB, or Gp32 mutants were constructed using a KOD-Plus-mutagenesis kit (Toyobo) with pST4-1 or pBAD33-gp32 as the template. The following primers were used for mutagenesis: YO-714 and YO-715 for pST4-13 (AbpA Q58A), YO-715 and YO-716 for pST4-14 (AbpA K60A), YO-902 and YO-903 for pST4-15 (AbpB K128A), YO-904 and YO-906 for pST4-16 (AbpB D208A), YO-905 and YO-906 for pST4-17 (AbpB E209A), and YO-938 and YO-939 for pBAD33-gp32 (C87A C90A). The DNA sequences of the constructed plasmids were confirmed through sequencing.

**TABLE 2:** Oligonucleotide sequences used in this study

Primer name	Sequence (5′–3′)
YO-714	GCGGTAAAAAGCACCGGCAAAACACG
YO-715	CAGGTAGCAAGCACCGGCAAAACACGTTGGAAC
YO-716	AACGAAGTCGATGTATCCATCAG
YO-819	GCGGTACCCGTAGAAATTATTCTTTCTC
YO-820	CTCTAGACTAAAATTTTAATTCATTCGCC
YO-899	GACAAGCTTTTAAAGGTCATTCAAAAGGTCATCCAG
YO-902	GCAGTGCAATCGTCGATTCATTGCTCGG
YO-903	TGCCCATGCTTGTAGGTGCACTCAG
YO-904	GCAGAGTTTTACAAGTTGGCGTTCCGAC
YO-905	GACGCGTTTTACAAGTTGGCGTTCCGAC
YO-906	AATGACGAAGAGGTCAATGTCGACG
YO-912	GCGAATTCACCATGTTTAAACGTAAATCTACTGCTGAAC
YO-924	GACGTCGACAAGCGCTATTGGTAATGGTACAATCGCGCG
YO-925	GACGCATGCTAACCTATTGTTTTAATGACAAATCAGAAC
YO-929	GCGAATTCACCATGTTTAAACGTAAATCTACTGCTGAAC
YO-930	GCCTGCAGTTAAAGATCATTCAAAAGGTCATCC
YO-931	GCGAATTCACCATGTTTAAACGTAAAAGTACCGCAGACC
YO-932	GCCTGCAGTTATAGACCAGCTAACAGGTCATCG
YO-938	GCCCCAGTAGCTCAATACATCAGTAAAAATGA
YO-939	AGAATCGTAATCACCATGGGTAGATG

### *E. coli* growth and CFU assay

*E. coli* cells were grown in a Luria–Bertani (LB) broth supplemented with ampicillin (AMP) (Nacalai Tesque, Kyoto, Japan), chloramphenicol (Nacalai Tesque), or both at 37°C. When the OD_660_ reached 0.3–0.5, 0.2% L-ara (FUJIFILM Wako Pure Chemical, Osaka, Japan) was added to express the protein from pBAD, and 1 µg/mL MMC (FUJIFILM), 0.1 µg/mL NFLX (Nacalai Tesque), 10 µg/mL BLM (Nacalai Tesque), 500 µg/mL hydroxyurea (Nacalai Tesque), 1 µg/mL 5-azacytidine (Nacalai Tesque), 2 µg/mL streptomycin (Nacalai Tesque), or phage T4 or φX174 was added. The cell density was monitored every 20 min using a biophotorecorder (TVS062CA; Advantec, Tokyo, Japan). Measurements were performed at least in triplicate, and similar results were obtained for each measurement. In the CFU assays, *E. coli* cells were grown in the LB broth supplemented with AMP at 37°C until the OD_660_ reached 0.4 and were harvested at 0, 2, and 4 h after adding MMC. The cells were diluted with phosphate-buffered saline (Nacalai Tesque), plated on LB-agar plates, and incubated at 37°C overnight. The colonies were counted, and the number of viable cells per 1 mL of the culture medium was calculated.

### Induction of Sp5 phages

MG1655-Sp5 (km^r^) cells harboring pBR322 or pST4-1 were grown in an LB medium supplemented with KM at 37°C until the OD_660_ reached approximately 0.4 and treated with 1.0 µg/mL MMC for 4, 6, or 8 h (35). Cell cultures were centrifuged at 8,000 *× g* for 3 min, and the supernatant was used for a plaque-forming assay. The phage solution was mixed with MG1655 as an indicator cell in soft agar containing LB medium, 0.3% agar, 1.5 µg/mL MMC, and 10 mM CaCl_2_. The mixture was poured onto LB-agar plates and incubated at 37°C overnight before counting the plaque-forming units.

### Sp5 phage lysogeny

MG1655-Sp5 (km^r^) cells were grown in an LB medium supplemented with KM at 37°C until the OD_660_ reached approximately 0.4 and treated with 1.0 µg/mL MMC for 8 h. Cell cultures were centrifuged at 8,000 *× g* for 3 min, and the supernatant was used as the Sp5 lysate. A 100 µL aliquot of the Sp5 lysate was mixed with 1 mL TY0807 cells (3–5 *×* 10^8^ cells) harboring pBR322, pST4-1, pST4-11, or pST4-12 and incubated for 1 h at 30°C. The mixture was diluted with phosphate-buffered saline, plated onto LB-agar plates supplemented with or without KM, and incubated at 37°C overnight. Colonies were counted, and the lysogeny rate was calculated by dividing the number of colonies yielded on LB plates containing KM by that yielded on LB plates without KM.

### AbpA and AbpB structure prediction and alignment

Three-dimensional AbpA (UniProt ID: P52127) and AbpB (UniProt ID: P52126) model structures were obtained from the AlphaFold Protein Structure Database (https://alphafold.ebi.ac.uk/) (36, 37). AbpA and AbpB structural homologs were searched on the Dali server using the AbpA and AbpB models as queries (http://ekhidna2.biocenter.helsinki.fi/dali/) (38). Superimposing the AbpA model to Cap4 (PDB ID: 6WAN [18]) and the AbpB model to human RNA helicase Ddp5 (PB ID: 3FHT [21]) was performed using the SUPERPOSE program (39). All protein structures were depicted using the open-source PyMOL version 1.7 software (Schrödinger, Inc., New York, NY, USA). Multiple amino acid sequence alignments were performed using Clustal Omega (40). Alignments were drawn using EsPript3 (41).

## SUPPLEMENTAL MATERIAL

Fig. S1 msphere.00372-23-s0001.tif Effect of T4 Gp32 or *E. coli* SSB on the growth of *E. coli* cells expressing AbpA, AbpB, or both.

Supplemental figure legends msphere.00372-23-s0002.docx Legends to Fig. S1 to S4.

Fig. S2 msphere.00372-23-s0003.tif Effect of DNA synthesis or translation inhibitors on the growth of *E. coli* cells expressing AbpAB.

Fig. S3 msphere.00372-23-s0004.tif Effect of *recB* or *recC* deletion on the growth of cells with or without AbpAB expression.

Fig. S4 msphere.00372-23-s0005.tif Effect of AbpAB expression on the lytic propagation of Sp5 phage.

ASM does not own the copyrights to Supplemental Material that may be linked to, or accessed through, an article. The authors have granted ASM a non-exclusive, world-wide license to publish the Supplemental Material files. Please contact the corresponding author directly for reuse.
