# Supplementary figures and images for: Phage single-stranded DNA-binding protein or host DNA damage triggers the activation of the AbpAB phage defense system
Source: mSphere. 2023 Oct 26;8(6):e00372-23. doi: 10.1128/msphere.00372-23 (PMC10732053; doi:10.1128/msphere.00372-23)

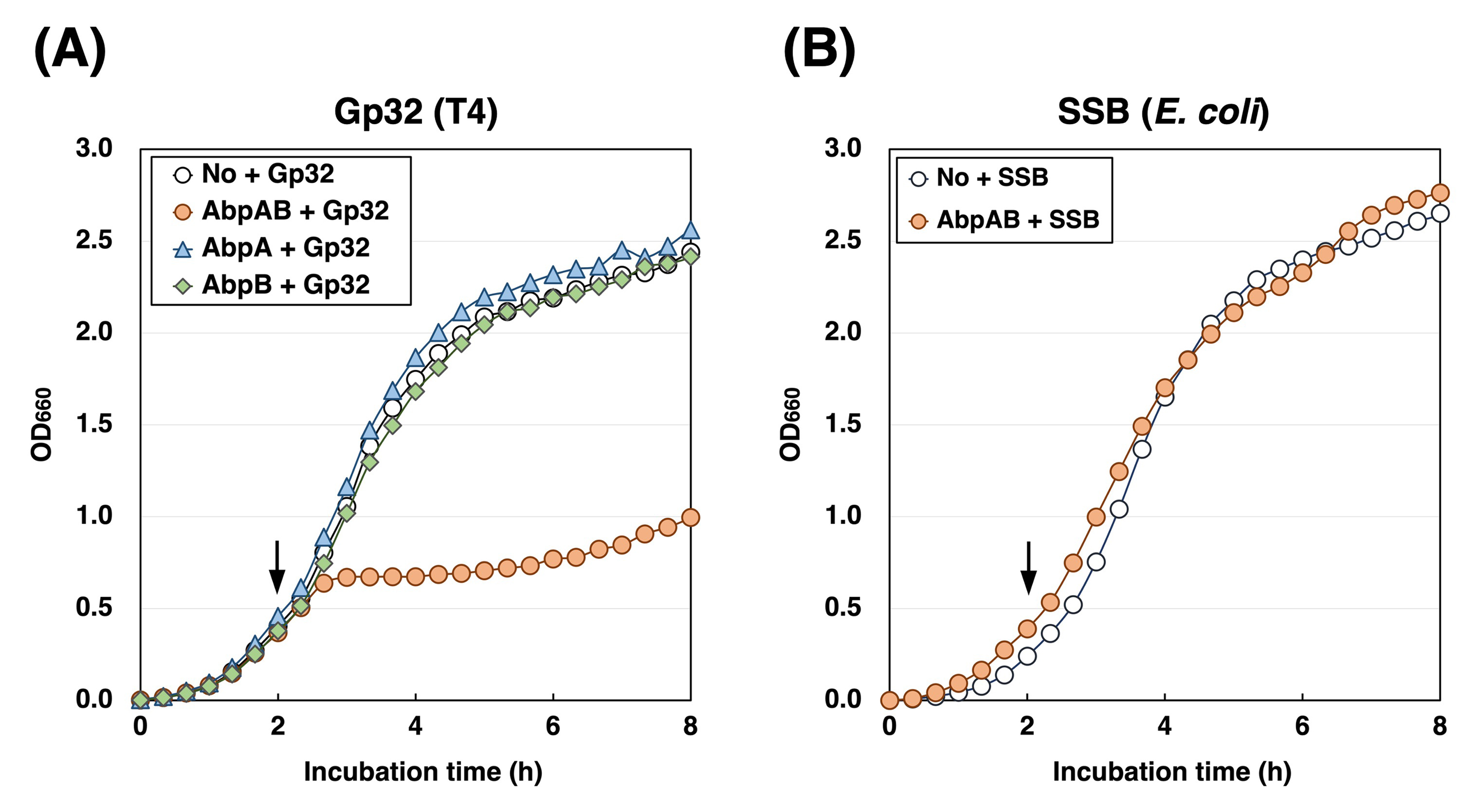

Supplement: Fig. S1 — Effect of T4 Gp32 or E. coli SSB on the growth of E. coli cells expressing AbpA, AbpB, or both. [file msphere.00372-23-s0001.tif]

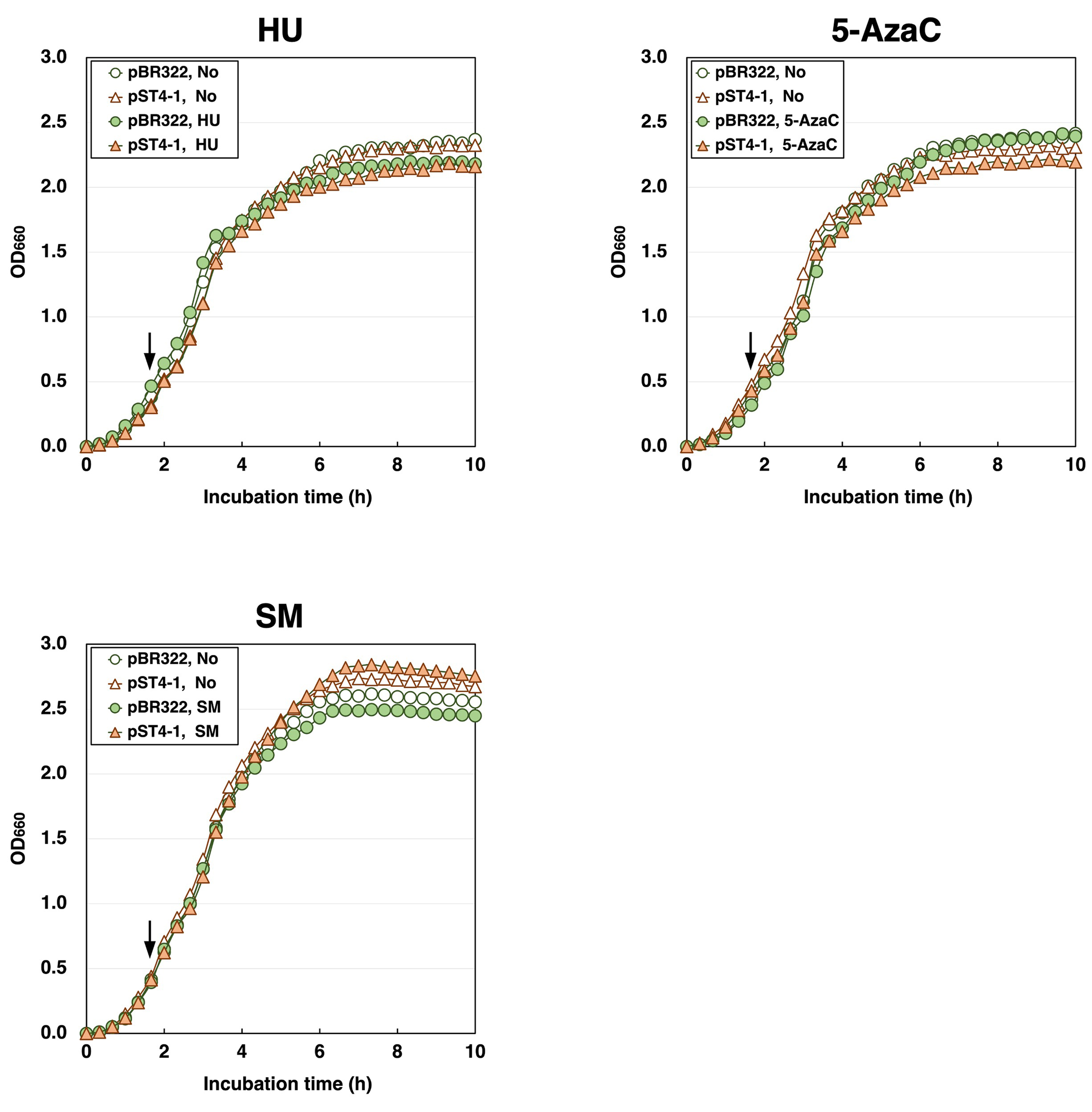

Supplement: Fig. S2 — Effect of DNA synthesis or translation inhibitors on the growth of E. coli cells expressing AbpAB. [file msphere.00372-23-s0003.tif]

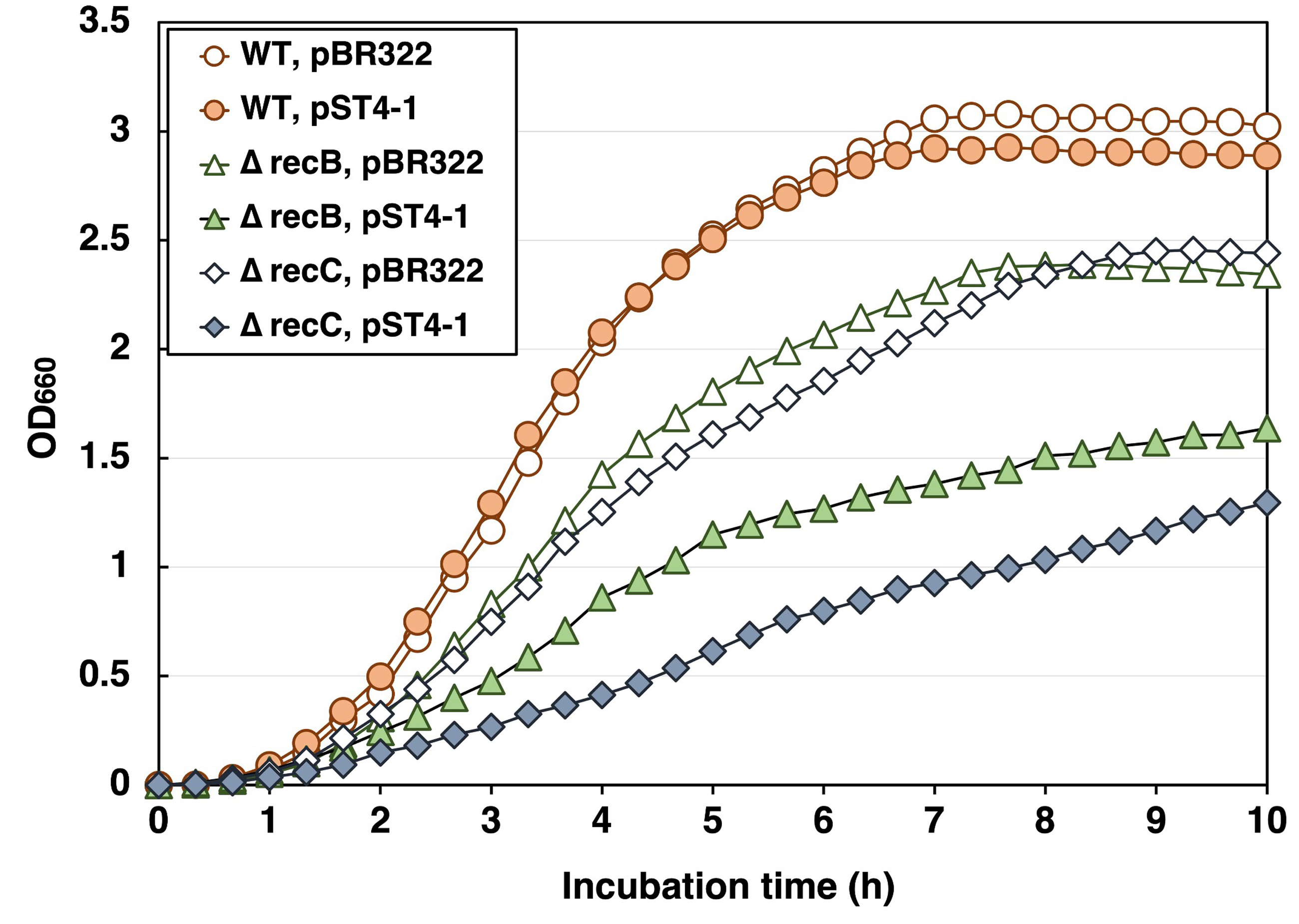

Supplement: Fig. S3 — Effect of recB or recC deletion on the growth of cells with or without AbpAB expression. [file msphere.00372-23-s0004.tif]

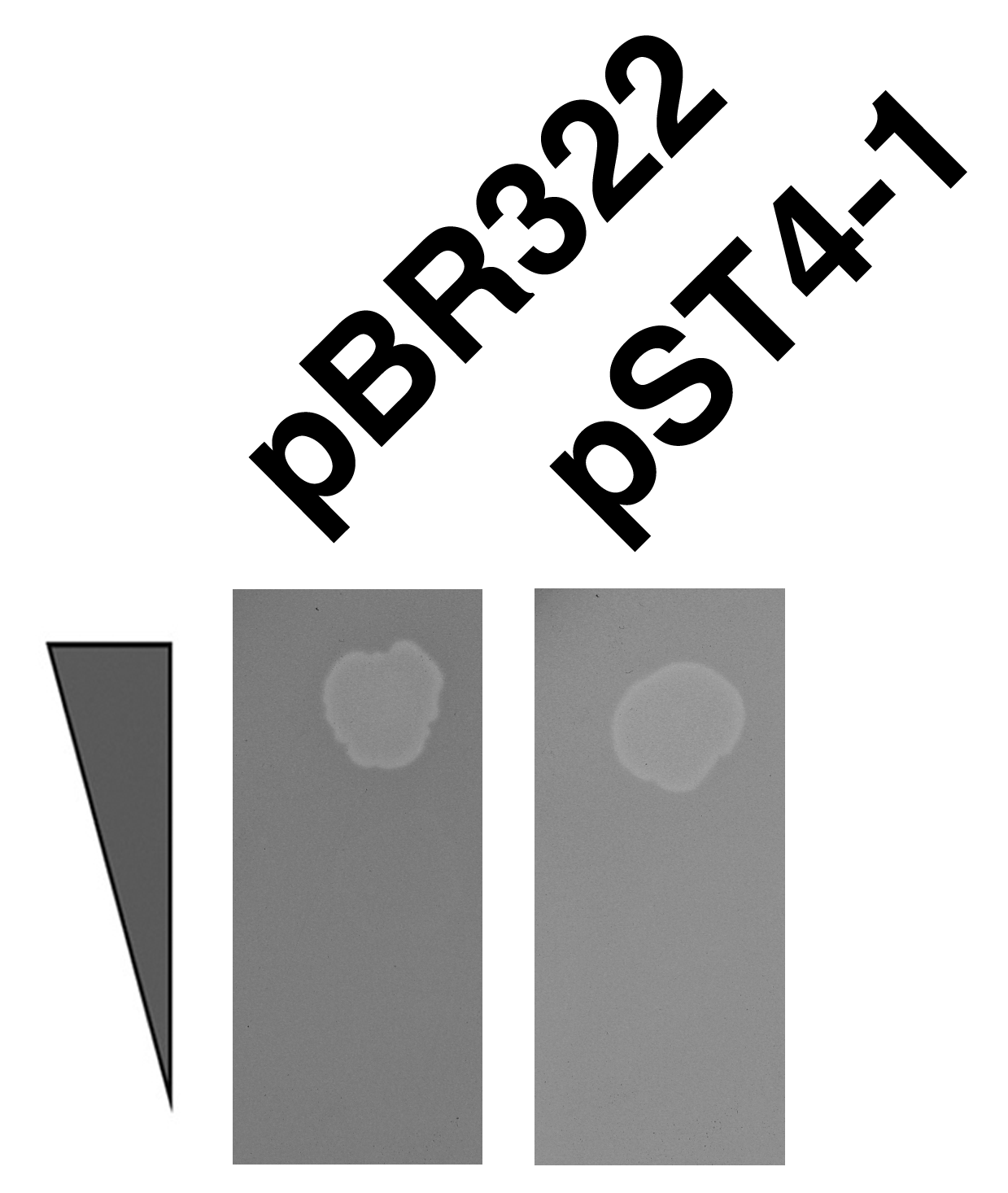

Supplement: Fig. S4 — Effect of AbpAB expression on the lytic propagation of Sp5 phage. [file msphere.00372-23-s0005.tif]
