# Supplementary material for: Phage single-stranded DNA-binding protein or host DNA damage triggers the activation of the AbpAB phage defense system
Source: mSphere. 2023 Oct 26;8(6):e00372-23. doi: 10.1128/msphere.00372-23 (PMC10732053; doi:10.1128/msphere.00372-23)
Supplement: Supplemental figure legends — Legends to Fig. S1 to S4. [file msphere.00372-23-s0002.docx]

Fig. S1 Effect of T4 Gp32 or *E. coli* single-strand DNA-binding protein (SSB) on the growth of *E. coli* cells expressing AbpA, AbpB, or both. (A) TY0807 cells harboring pBAD33-gp32 plus pBR322 (No + Gp32), pST4-1 (AbpAB + Gp32), pST4-12 (AbpA + Gp32), or pST4-11 (AbpB + Gp32) were treated with L-ara when the OD_660_ reached approximately 0.4. (B) TY0807 cells harboring pBR322 and pBAD33-ssb (No + SSB) or pST4-1 and pBAD33-ssb (AbpAB + SSB) were treated with L-ara when the OD_660_ reached approximately 0.3.

Fig. S2 Effect of DNA synthesis or translation inhibitors on the growth of *E. coli* cells expressing AbpAB. TY0807 cells harboring pBR322 or pST4-1 were treated with or without hydroxyurea (HU), 5-azacytidine (5-AzaC), or streptomycin (SM) when the OD_660_ reached approximately 0.5.

Fig. S3 Effect of *recB* or *recC* deletion on the growth of cells with or without AbpAB expression. BW25113 (WT), JW2788 (∆*recB*), and JW2790 (∆*recC*) cells harboring either pBR322 or pST4-1 were cultured.

Fig. S4 Effect of AbpAB expression on the lytic propagation of Sp5 phage. MG1655-Sp5 (km^r^) cells were grown in an LB medium supplemented with KM at 37 °C until the OD_660_ reached approximately 0.4. Cells were treated with 1.0 µg/mL MMC for 8 h. Cell cultures were centrifuged at 8,000 ×*g* for 3 min, and the supernatant was used for a plaque-forming assay. An Sp5-containing suspension at a 1:10 dilution was spotted onto plates seeded with MG1655 cells harboring pBR322 or pST4-1 as indicators in soft agar containing LB medium, 0.3% agar, 0.2 µg/mL MMC, and 10 mM CaCl_2_. The plates were incubated overnight at 30 °C.
